# Supplementary material for: DMRforPairs: identifying Differentially Methylated Regions between unique samples using array based methylation profiles
Source: BMC Bioinformatics. 2014 May 15;15:141. doi: 10.1186/1471-2105-15-141 (PMC4046028; doi:10.1186/1471-2105-15-141)
Supplement: Additional file 2 — DMRforPairs output for the comparison of A431-MCF7 and NA17018-NA17105. Please start from the HTML files in each folder. Available via the BMC Bioinformatics website. [file 1471-2105-15-141-S2.zip › 1394847754114233_MOESM2_ESM/A431_MCF7/figures/27776.pdf]

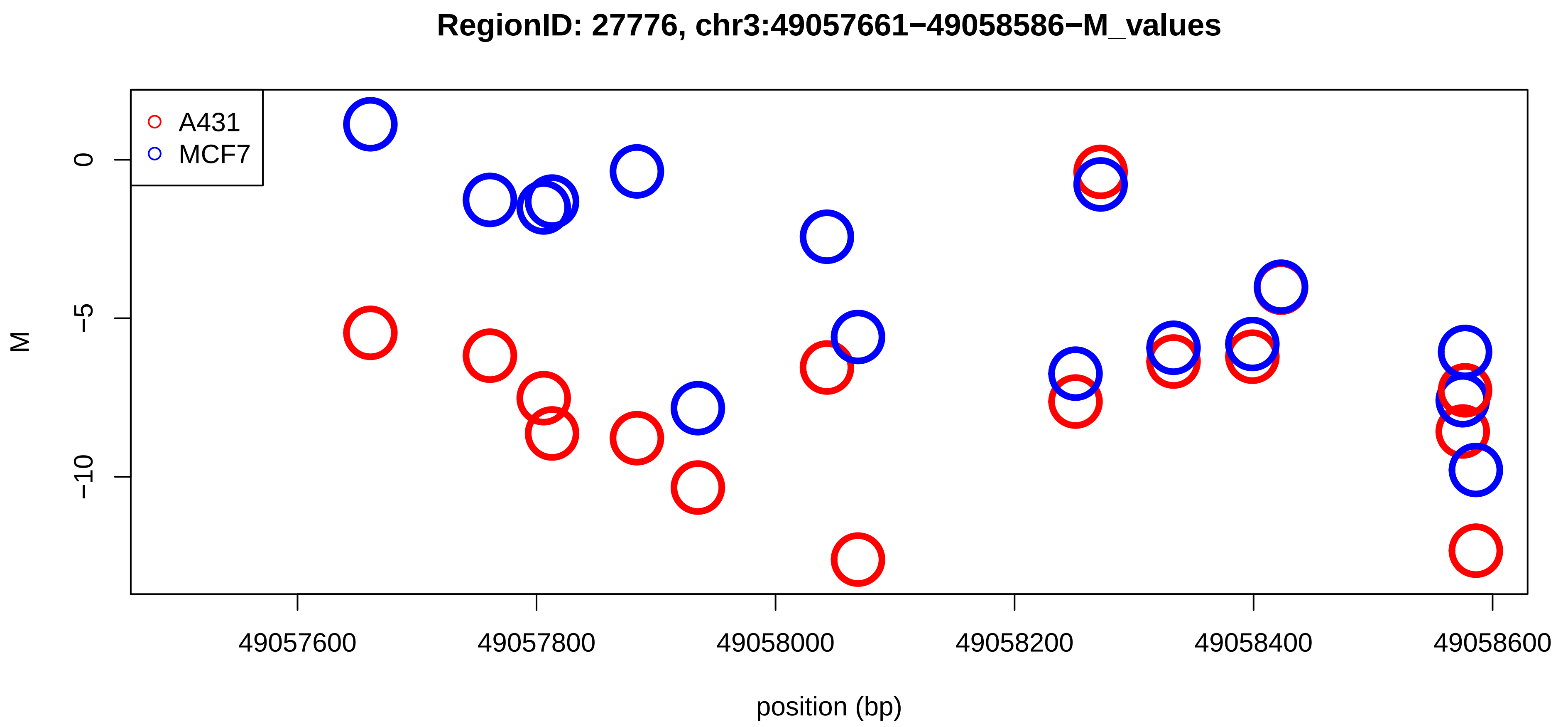
RegionID: 27776, chr3:49057661−49058586−M_values
A431
MCF7
0
−5
−10
M
49057600
49057800
49058000
49058200
49058400
49058600
position (bp)

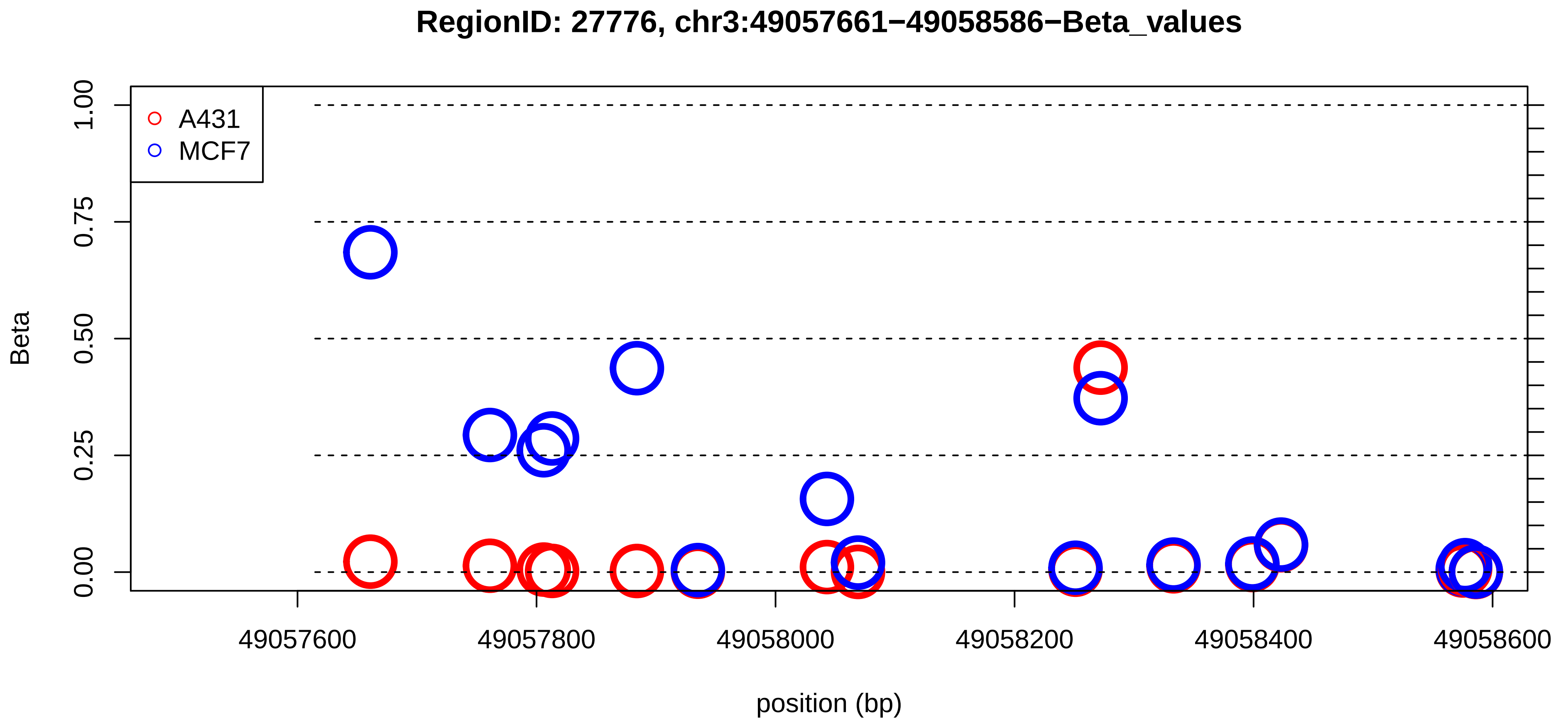
RegionID: 27776, chr3:49057661−49058586−Beta_values
A431
MCF7
1.00
0.75
0.50
0.25
0.00
Beta
49057600
49057800
49058000
49058200
49058400
49058600
position (bp)

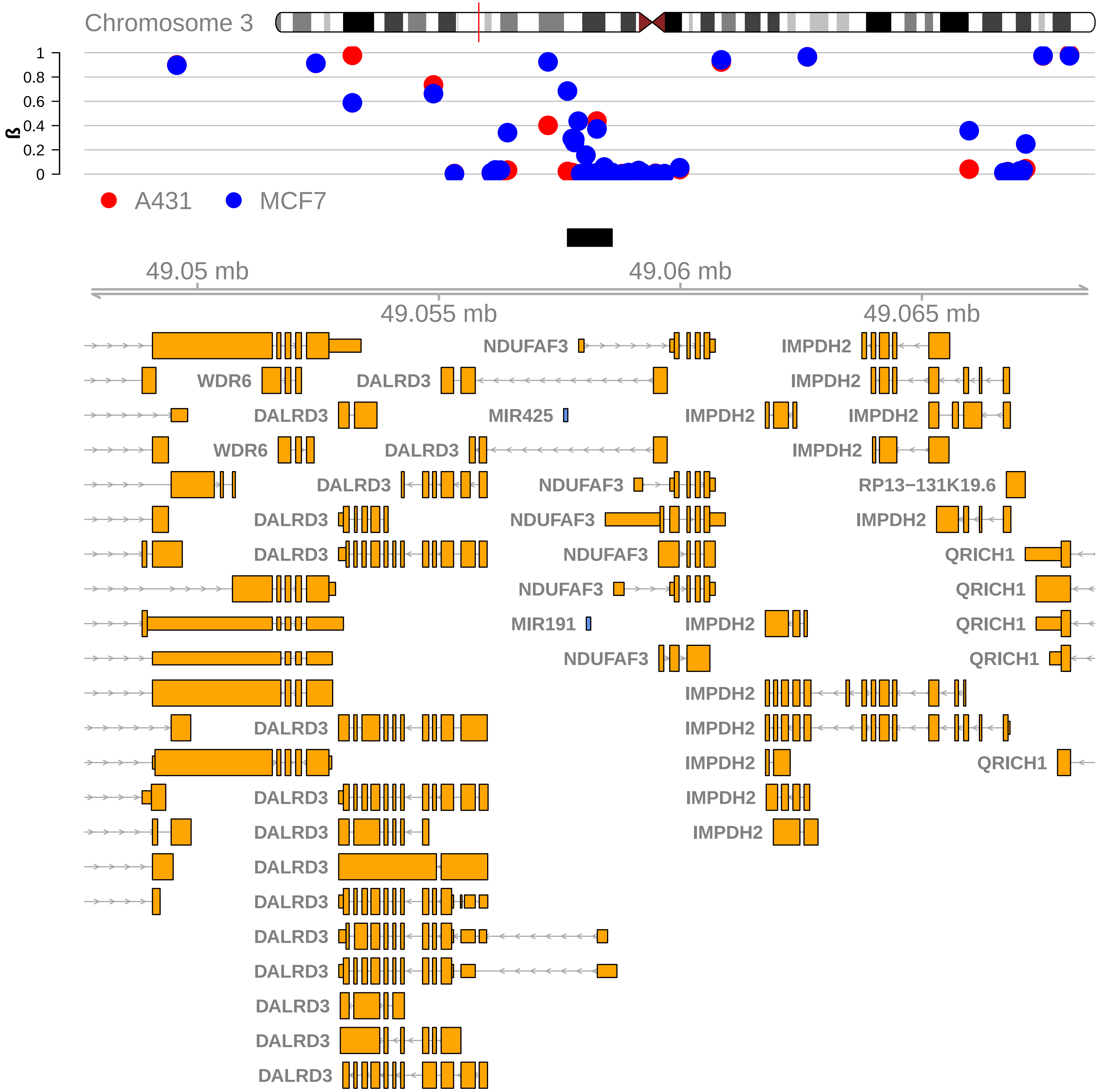

Chromosome 3
ß
1
0.8
0.6
0.4
0.2
0
A431
MCF7
49.05 mb
49.06 mb
49.055 mb
49.065 mb
NDUFAF3
IMPDH2
WDR6
DALRD3
MIR425
RP13−131K19.6
QRICH1
MIR191
